# Superior orbitectomy and chemotherapy in a dog with frontal sinus squamous cell carcinoma: a case report and review of the literature

**DOI:** 10.1002/ccr3.889

**Published:** 2017-03-08

**Authors:** Andrea Steinmetz

**Affiliations:** ^1^ Department of Small AnimalsUniversity of LeipzigAn den Tierkliniken 23D‐04103LeipzigGermany

**Keywords:** Chemotherapy, dog, frontal sinus squamous cell carcinoma, pFS‐SCC, superior orbitectomy

## Abstract

A superior orbitectomy can be a challenging but accomplishable surgical option in dogs with a tumor which involves the dorsal bony part of the orbit. The procedure described in this report can be vision‐sparing and life prolonging even in a case of an aggressive growing frontal sinus squamous cell carcinoma.

## Introduction

Squamous cell carcinoma (SCC), which primarily originates from the mucosal lining of the frontal sinus, is a rare disease in humans as well as in animals 1. It was first described in humans in 1907 by Prawssud 2. Only 0.009–0.03% of all head and neck cancers in men are primary frontal sinus‐SCC (pFS‐SCC) 3, 4. Mine et al. (5) studied 32 patients with sinonasal malignant tumors who underwent craniofacial resection and found that involvement of the frontal sinus and involvement of the orbit and detection of a SCC were prognostic factors for poor overall survival time 5.

Treatment of frontal sinus tumors in humans is inconsistent 1. Radical surgery or surgery in combination with radiation is the first line of therapy 4, 6, 7, 8, 9. One study indicated that radical surgery alone is the treatment of choice for therapy of pFS‐SCC 10. Local control and disease‐free survival were adversely influenced by incomplete resection 5.

Complete resection and adjuvant therapy (chemo‐ or radiotherapy) are the recommendations of another study for the treatment of this sort of tumor. However, it is unclear whether the use of chemotherapy and/or radiotherapy can improve the prognosis of pFS‐SCC in humans.^1^.

In dogs and cats cranial and orbital tumors pose diagnostic and therapeutic challenges. Most tumors located in the periorbital region are malignant 11, 12, 13, 14. Tumors of the nasal cavity and paranasal sinuses together account for approximately 1% of all neoplasms in dogs 15. ata regarding incidence of pFS‐SCC in animals have not yet to be provided, and pFS‐SCC in dogs was described in only a few case reports or small case series 16, 17, 18. Vascellari et al. described pFS‐SCC in an addax 19. PFS‐SCC can invade the orbit, the cranium and nasal sinus 16.

Precise recommendations for the treatment of pFS‐SCC in animals exist.

Without therapy, eight dogs with SCC of the nasal cavity and frontal sinus had a median survival time of 3 months 17. Radiation and surgery for the treatment of nasal SCC 20 as well as high dose brachytherapy have been recommended for the treatment of intranasal tumors 21 in dogs. Adjuvant irradiation is recommended in cases of incomplete resection of SCC 22. PFS‐SCC was medically treated in three dogs using piroxicam in combination with carboplatin or toceranib 16.In this case report, a pFS‐SCC was diagnosed and treated with radical surgery and subsequent chemotherapy.

## Case Report

A 9.5‐year‐old spayed female Small Munsterlander (body weight 14 kg) was presented with a facial deformity limited to the left frontal sinus region. A mass (about 3 × 3 × 3 cm) had developed over a three‐month period. The owners described sneezing and sniffing before and after visibility of the mass. Apart from that, abnormal clinical signs were not noted.

### Clinical examination and findings

#### General clinical examination

**Figure 1:**
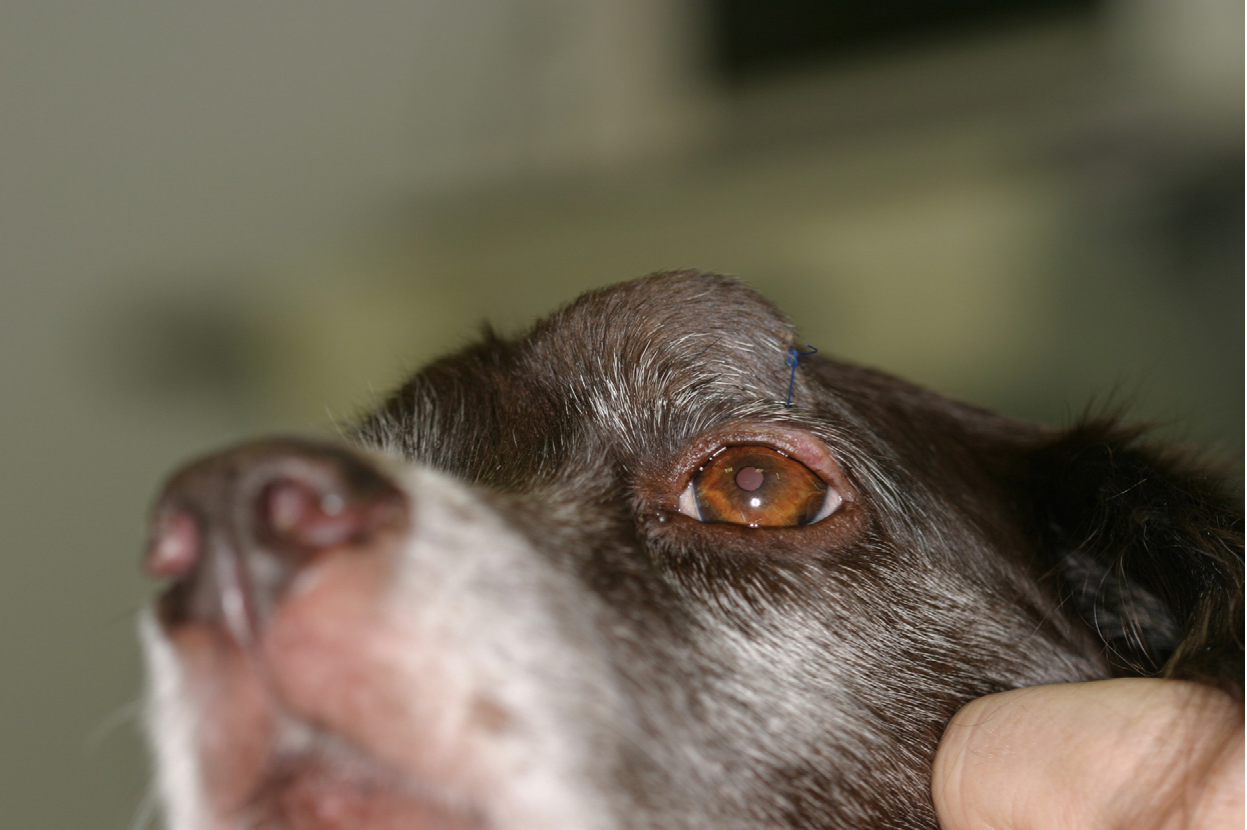
A solid mass of about 3 × 3 × 3 cm could be seen dorso‐medially of the left eye. (Picture made after biopsy).

The dog was alert and in fairly good body‐condition. A solid mass with a diameter of 3 cm could be observed dorsomedially to the left eye (Fig. 1). The mass was fixed in position and located in the left frontal sinus region. The rest of the physical examination including palpation of the regional lymphnodes showed no abnormalities.

#### Ophthalmic examination

A complete ophthalmic examination was performed including biomicroscopy (SL 14, Kowa Company Ltd, Tokyo/Japan), indirect ophthalmoscopy with 30‐ and 20‐diopter condensing lenses (Omega 200, Heine, Herrsching, Germany), and tonometry (TonoVet, Icare, Espoo, Finland). The left eye showed minimal proptosis with a slight ventro‐lateral deviation.

The eyelids, conjunctiva, cornea, and anterior chamber were normal bilaterally. Both lenses showed minor fiberglass/crystal‐like nuclear cataracts. Examination of the fundus revealed bilateral local and symmetrical chorioretinal hypoplasia. The intraocular pressure was 8 and 6 mm Hg in the right and left eye, respectively.

### Further investigations

For determination of appropriate therapeutic options, blood chemistry (BC), complete blood cell count (CBC), abdominal ultrasound, and computed tomography (CT) examinations of the head and thorax and biopsies of the mass were obtained. BC and CBC were found normal. Abdominal ultrasonography did not reveal discernible abnormalities.

#### Computed tomography

**Figure 2:**
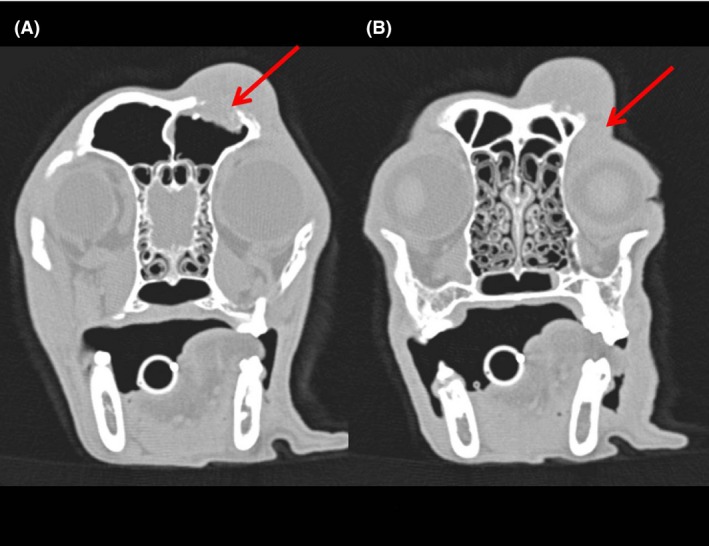
Computed tomography. Transversal plane. Focally extensive lysis and erosion of the bone. (A) Extension of the mass into the frontal sinus (red arrow). (B) Mild extension of the mass into the orbit (red arrow).

Anesthesia for the CT examination and biopsy was induced intravenously with 0.5 mg/kg diazepam (Faustan^®^, Temmler Pharma, Marburg, Germany) and 0.5 mg/kg levomethadone in a fixed combination with 0.025 mg/kg fenipramide (L‐Polamivet^®^, Intervet, Unterschleißheim, Germany). Anesthesia was maintained via endotracheal intubation with 1% isoflurane (Isofluran CP, CP Pharma, Burgdorf, Germany) dissolved in oxygen at a flow rate of 10 mL/kg/min. CT findings (six‐slice helical CT, Philips Brilliance 8000 Mx, Phillips Healthcare Hamburg, Germany) of the head included an isoattenuating (to surrounding musculature) soft tissue mass at the zygomatic process of the frontal bone, which caused lysis and erosion of the bone and extended into the frontal sinus and the orbit (Fig. 2). The axial frontal bone edge of the eroded area was thickened and mildly lobulated in the ventral area. Apart from these no further abnormalities of the neuro‐ and splanchnocranium were found. A thoracic CT examination revealed two nodules with a diameter of 2 mm within the right lung, each in the cranial and caudal lobe respectively.

#### Histopathologic examination

**Figure 3:**
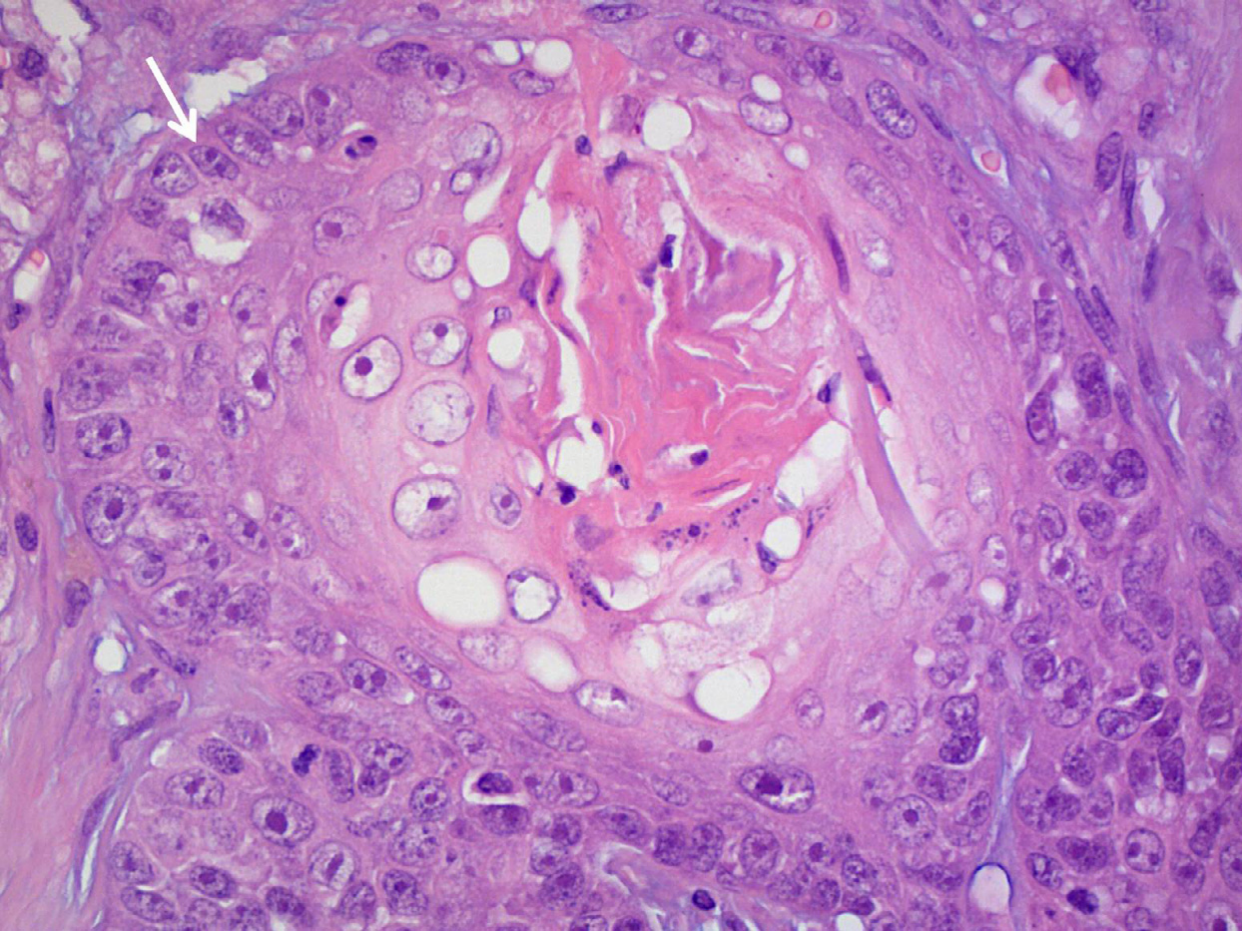
Squamous cell carcinoma. HE staining. Magnification 40x: Big cells with marked anisonucleosis, polymorphism of the nucleus, and marked nucleoli. Mitosis (white arrow).

The biopsy was performed in the same anesthesia. After transection of the skin over the tumor, 1 × 1 × 1 cm of the mass was excised and fixed in 4% formalin. Biopsy results confirmed squamous cell carcinoma of the frontal sinus (Fig. 3). The TNM stage for canine nasosinal tumors 22 in this dog at this time was T 3, N0 and questionable in M.

### Therapy and results

#### Surgical procedure

The owners only requested excision of the mass and did not wish any further diagnostic procedures pertaining to suspected incidental findings within the thorax due to the good general condition of the dog. Surgery was performed a week after the initial work‐up using the same anesthetic protocol as described above. A superior orbitectomy was chosen to excise the mass with a margin of 2 cm.

**Figure 4:**
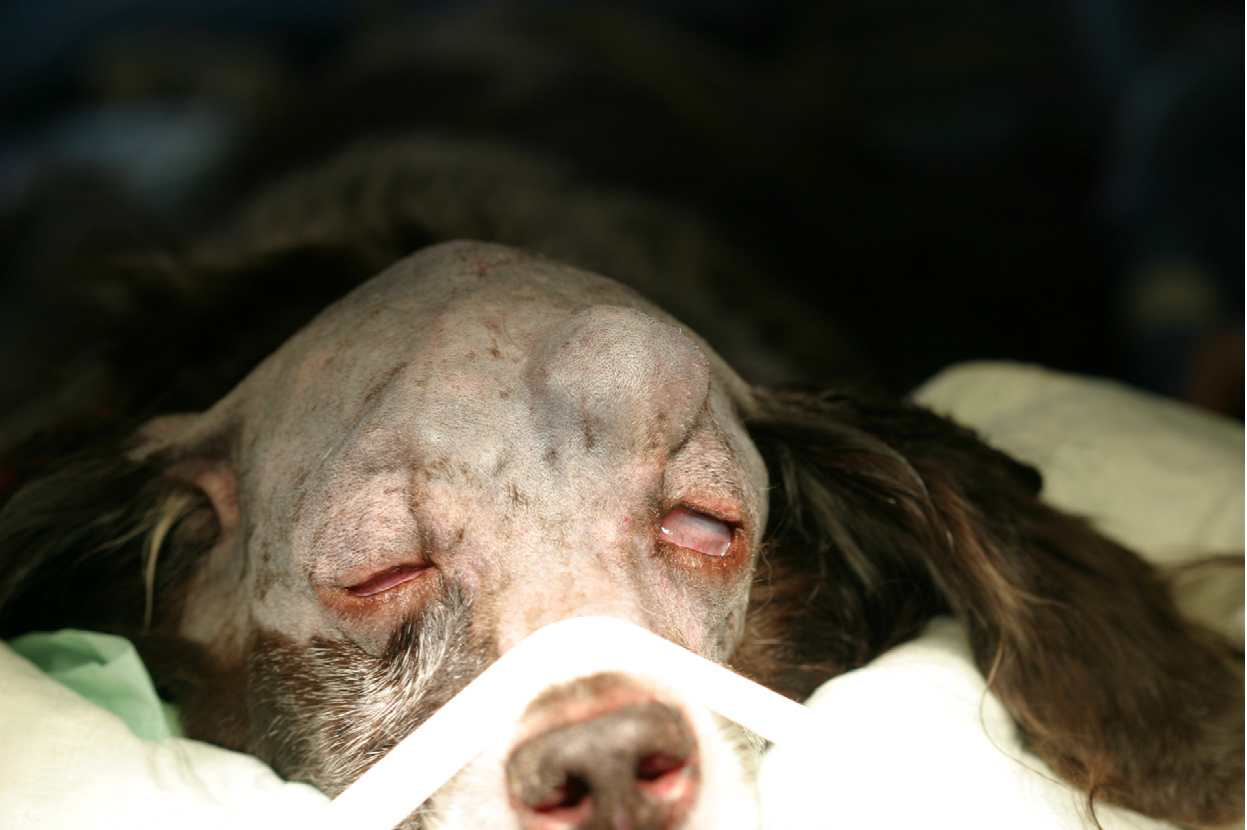
Positioning of the dog in sternal recumbency with the head in a vacuum pillow.

First, the surgical field (including the left eyelid) was clipped and prepared aseptically for surgery, and the dog was positioned in sternal recumbency (Fig. 4).

**Figure 5:**
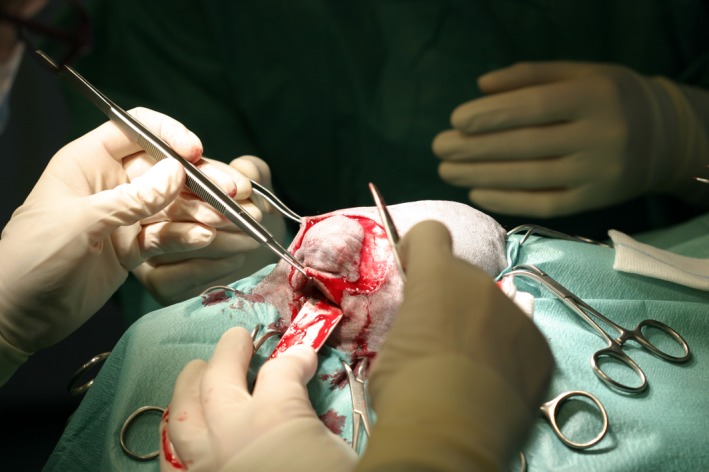
Skin incisions were made with a scalpel blade size 22. Margins of resected skin: The medial incision was made parallel to and approximately 1 cm to the right of the midline at the level of the zygomatic process and extended 5 cm. The caudal margin, which was perpendiculary oriented to the first ran 2 cm caudal to the zygomatic process of the frontal bone. Care was taken to leave the left eye, optic nerve, and retrobulbar muscles intact.

**Figure 6:**
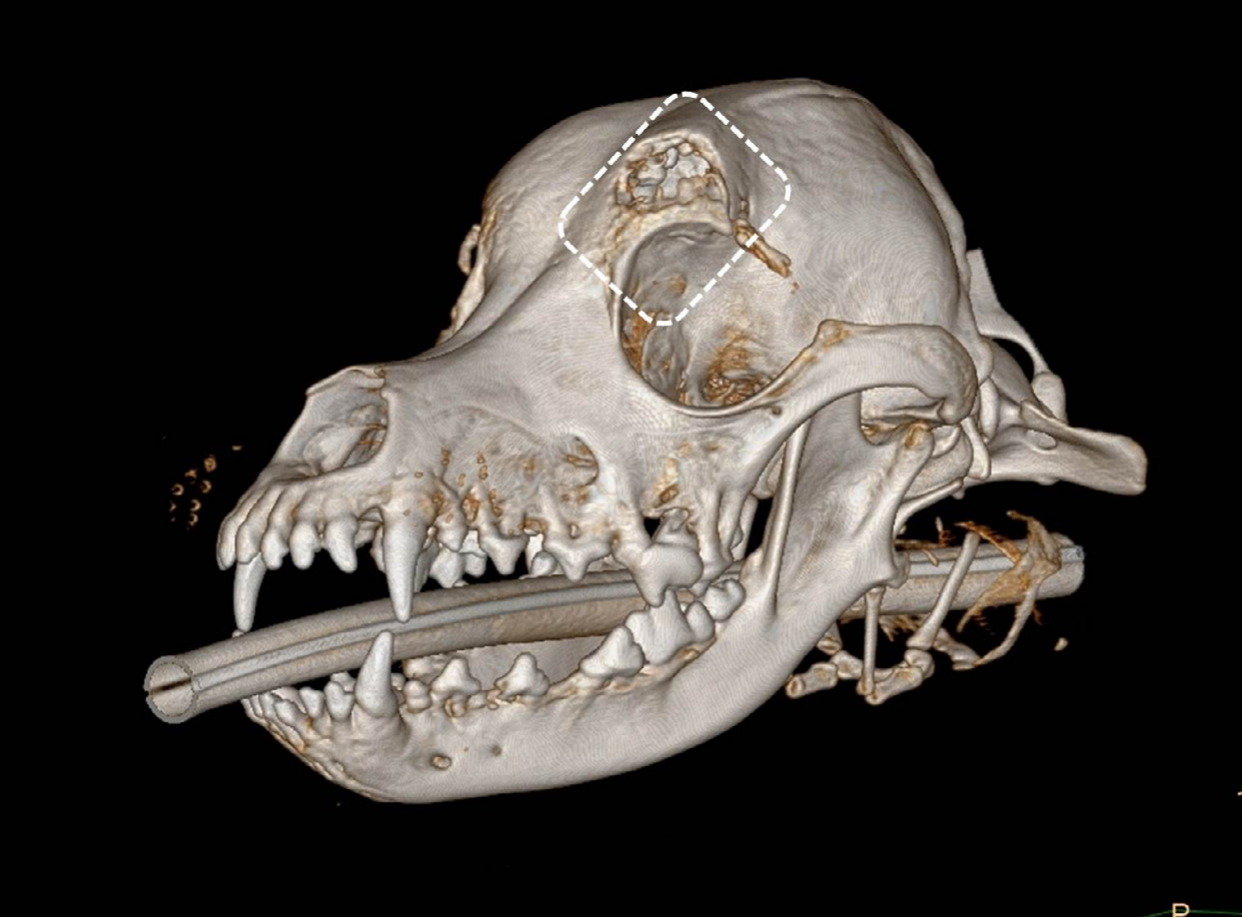
Computed tomography. 3‐D. Resected bones: part of the frontal bone, including the zygomatic process and medial part of the orbit.

Next, skin and subcutaneous muscle were cut sharply as shown in Figure 5. The lateral and rostral end points of the lines included a major part of the dorsal eyelid of the left eye (Fig. 5). In a further step the temporal muscle was divided with a scissor in order to expose the underlying bone. Thereafter, the removal of the affected bone parts skull (Fig. 6) was performed with an oscillating saw (Compact Air Drive^®^, Saw blade 70/49 × 20 × 0.6/0.4 mm; Saw Attachment with variable deflection; Synthes inc., West Chester, PA). In order to leave the left eye, optic nerve and retrobulbar muscles intact, extreme care was required.

**Figure 7:**
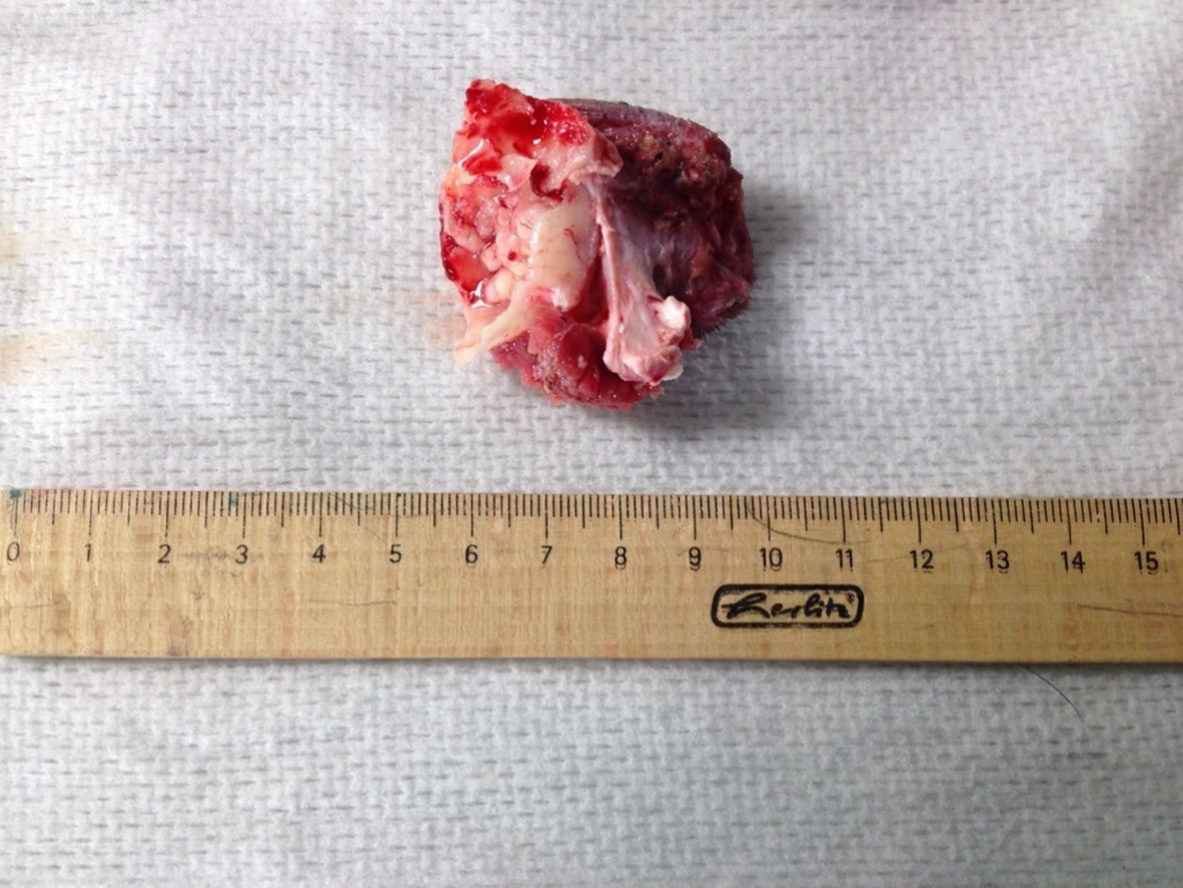
Removed tissue.

**Figure 8:**
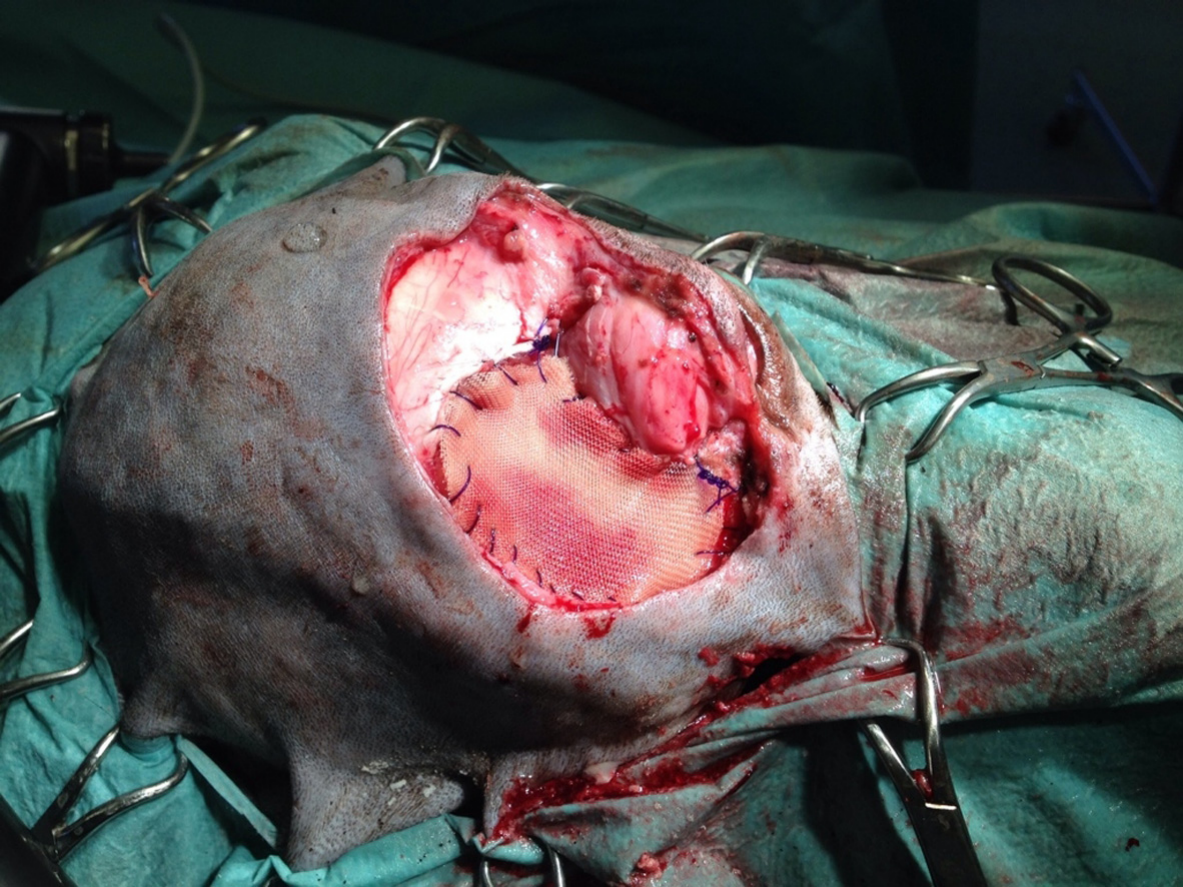
Mesh was sutured into the defect to close the sinus and to stabilize the orbital content, and to reach stability.

**Figure 9:**
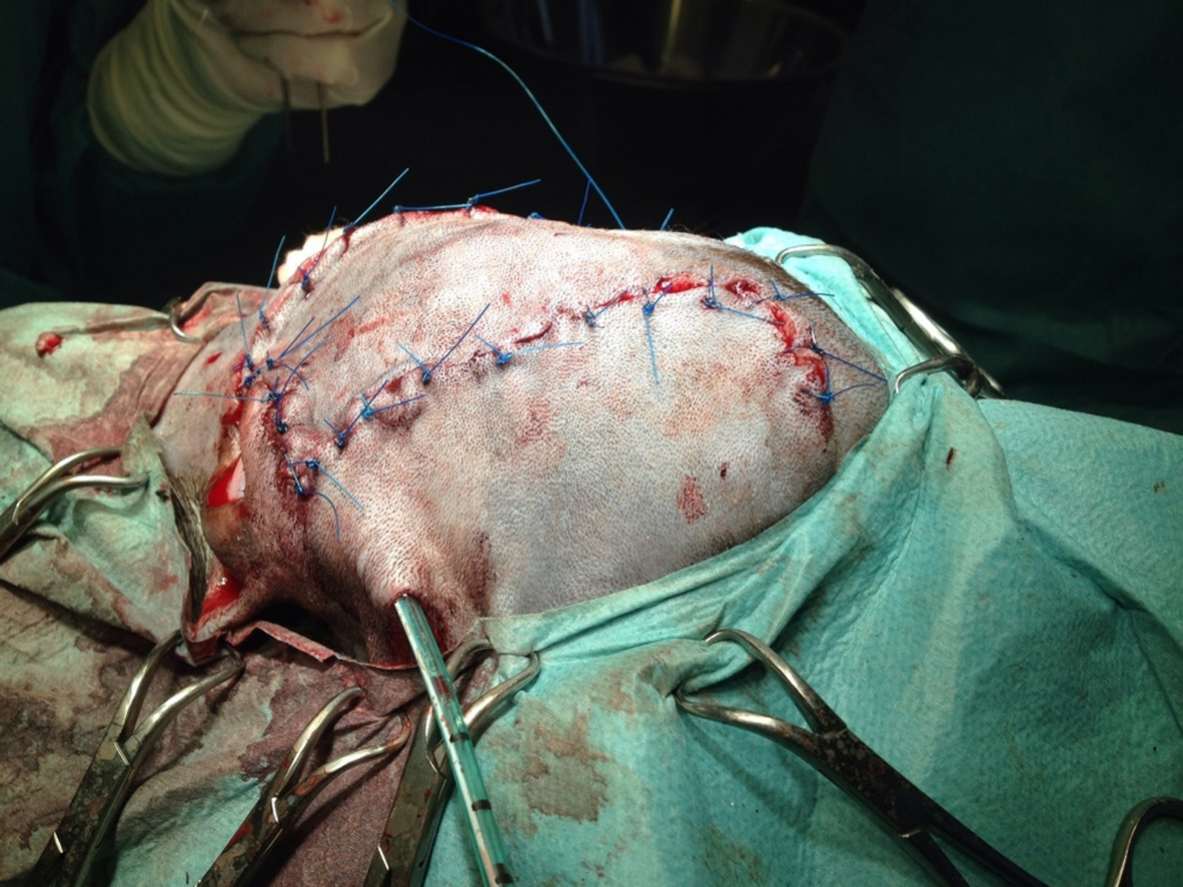
Advancement flap which was as broad but twice as long as the defect.

**Figure 10:**
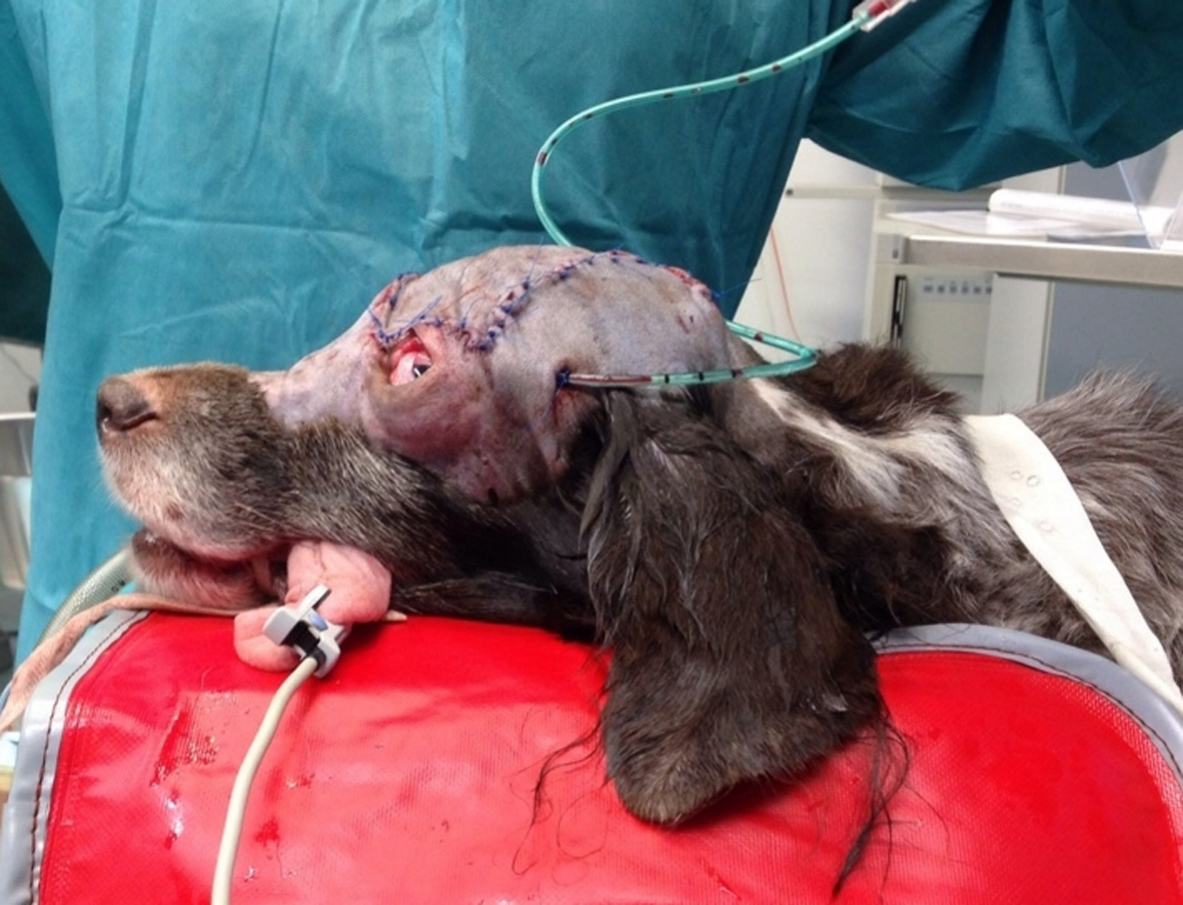
Active drainage system.

Due to the removal of the mass (Fig. 7), the left frontal sinus was exposed, and the medial part of the orbit and a major part of the dorsal eyelid were absent. Four tumor bed biopsies were taken. Afterward four layers of an artificial Polyglactine mesh (Vicryl^®^; Ethicon Johnson‐Johnson, Norderstedt, Germany) were sutured into the defect (Fig. 8). Subsequently, the skin of the dorsal eyelid was reconstructed using single pedicle advancement flap (Fig. 9). The subcutaneous layer and skin were closed with 3/0 poliglecaprone (Monocryl^®^, Ethicon Johnson‐Johnson, Norderstedt, Germany) and 4/0 polyamide (Ethilon^®^; Ethicon Johnson‐Johnson, Norderstedt, Germany) respectively using a simple interrupted suture pattern. Finally, to avoid seroma development an active drainage system (redon drain 12 charrière, 30 mL‐suction bag, Walter Veterinär‐Instrumente e.K., Baruth, Germany) was inserted into the left temporal region and placed beneath the flap (Fig. 10). The drain remained in place for 3 days.

The postoperative treatment involved 20 mg/kg BID amoxicillin/clavulanic acid (Synulox^®^, Pfizer AG, Freiburg, Germany) administered orally for 10 days, 4 mg/kg SID carprofen (Rimadyl^®^, Pfizer AG, Freiburg, Germany) administered for 3 days and topical eye drops (Gentamicin POS^®^, Ursapharm, Saarbrücken, Germany) administered five times a day for 10 days. Eye drops (Vislube^®^, TRB Chemedica AG, Haar, Germany) were administered four times a day for 2 months to ensure the moisturization of the conjunctiva and cornea. An Elizabethan collar prevented self‐inflicted trauma. The day after the surgery the habitus of the dog was found normal. He was discharged three days postoperatively after the drain removal. Postoperatively, the dog showed a slight ventro‐lateral deviation of the left globe, and he was able to close his eye despite an iatrogenic ectropium of the upper eyelid.

Histopathological examination confirmed the diagnosis of Squamous cell carcinoma. The SCC was moderately differentiated: in some areas well– in other areas poorly. Cytoplasm of the tumor cells stained strongly positive for pancytokeratin.

The histopathological examination revealed tumor cell free in 10 sections and clear medial, caudal and lateral tumor bed biopsies.

**Figure 11:**
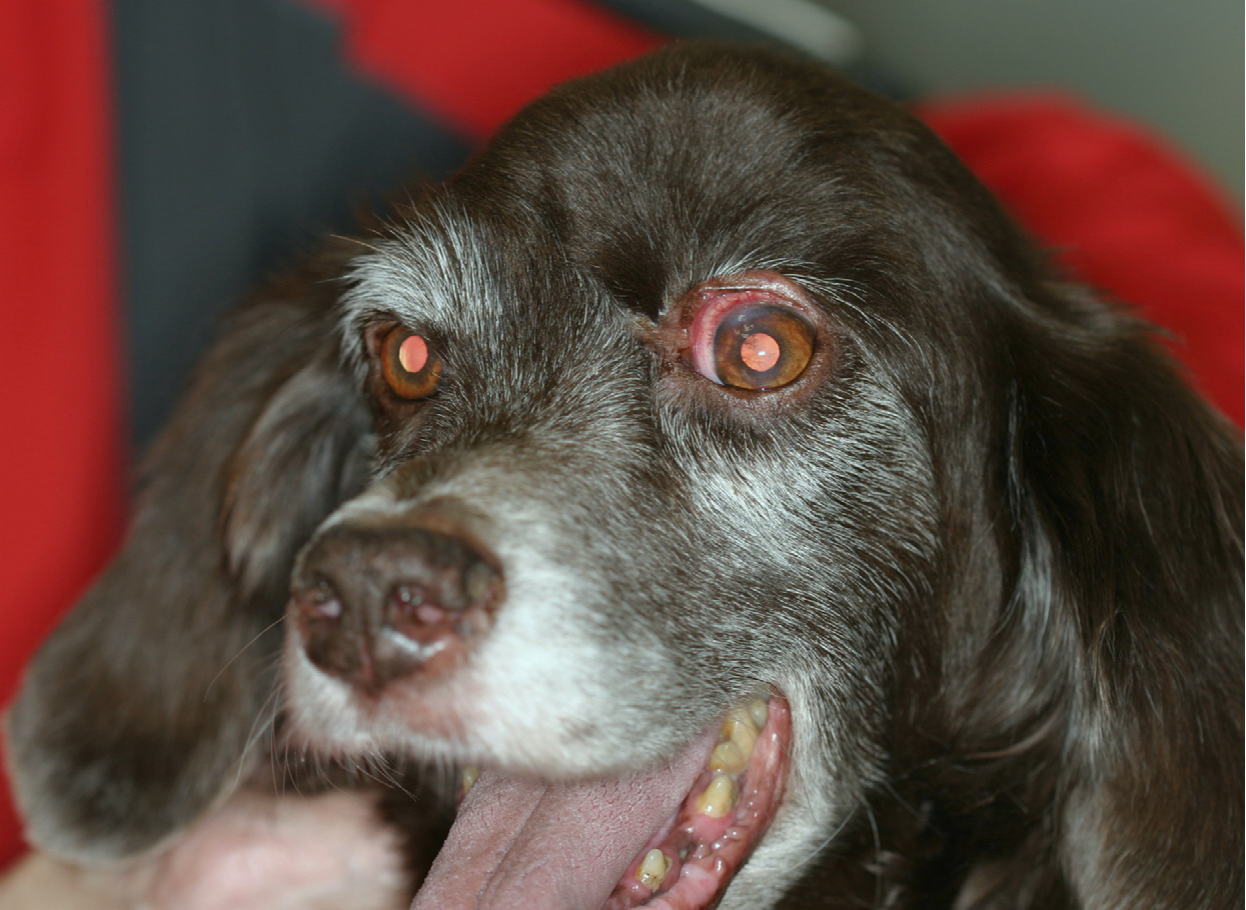
Left eye with a slight ectropium of the dorsal eyelid.

At the three‐month and five‐month postoperative follow‐up, the patient seemed comfortable, showed normal vision in the left eye and an acceptable cosmetic result of the surgical site (Fig. 11).

The dog was disease free about 9 month and presented again twelve months after surgery due to nasal discharge over the previous 3 months. The general physical and the local examination showed no changes compared with the follow‐up examination 7 months before. There was no visible sign of tumor regrowth at this time. However, follow‐up CT examinations of the head and thorax were recommended and performed under the same anesthetic protocol as previously described.

**Figure 12:**
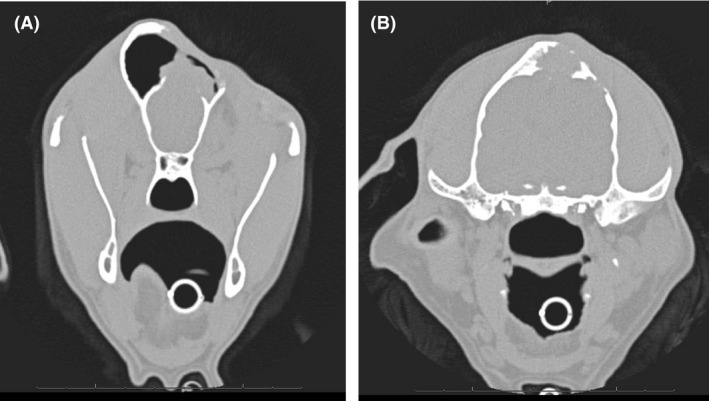
Computed tomography. Transversal plane. 12 month after surgery. (A) Soft tissue mass in the frontal sinus. (B) Destruction of the cranial bone.

The CT revealed againa soft tissue mass in the frontal sinus, as well as progressive bone destruction (Fig. 12). The small pulmonary nodules were unaltered compared to the investigation performed twelve months previously.

Due to disappearing of the conchae, rhinoscopy, and biopsy were performed. Rhinoscopy revealed atrophy of the conchae. Histopathological examination revealed an aseptic rhinitis.

The new biopsy of the mass in the frontal sinus showed SCC again, thus confirming local recurrence.

#### Chemotherapy

Consequently chemotherapy was initiated with carboplatin 300 mg/m^2^ (Carboplatin Omnicare, Omnicare Holding GmbH & Co. KG, Unterfoehring, Germany) every 3 weeks with six repetitions. Meloxicam 0, 1 mg/kg SID (Metacam^®^, Boehringer Ingelheim Vetmedica GmbH, Ingelheim, Germany) was added to carboplatin. The chemotherapy was well tolerated by the dog.

**Figure 13:**
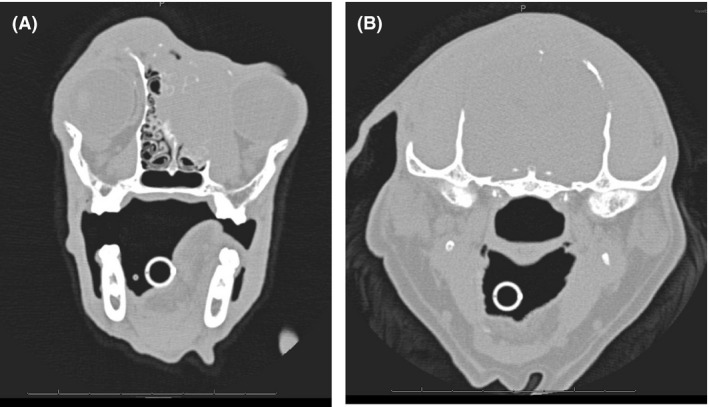
Computed tomography. Transversal plane. Five month after initiating chemotherapy. (A) Further progression of the mass with entering the orbit. (B) Destruction of the bone and suspected involvement of the brain.

For the purpose of evaluation of the response to treatment CT examination of the head was performed 5 months after initiating chemotherapy but further progression of the mass has to be stated (Fig. 13).

**Figure 14:**
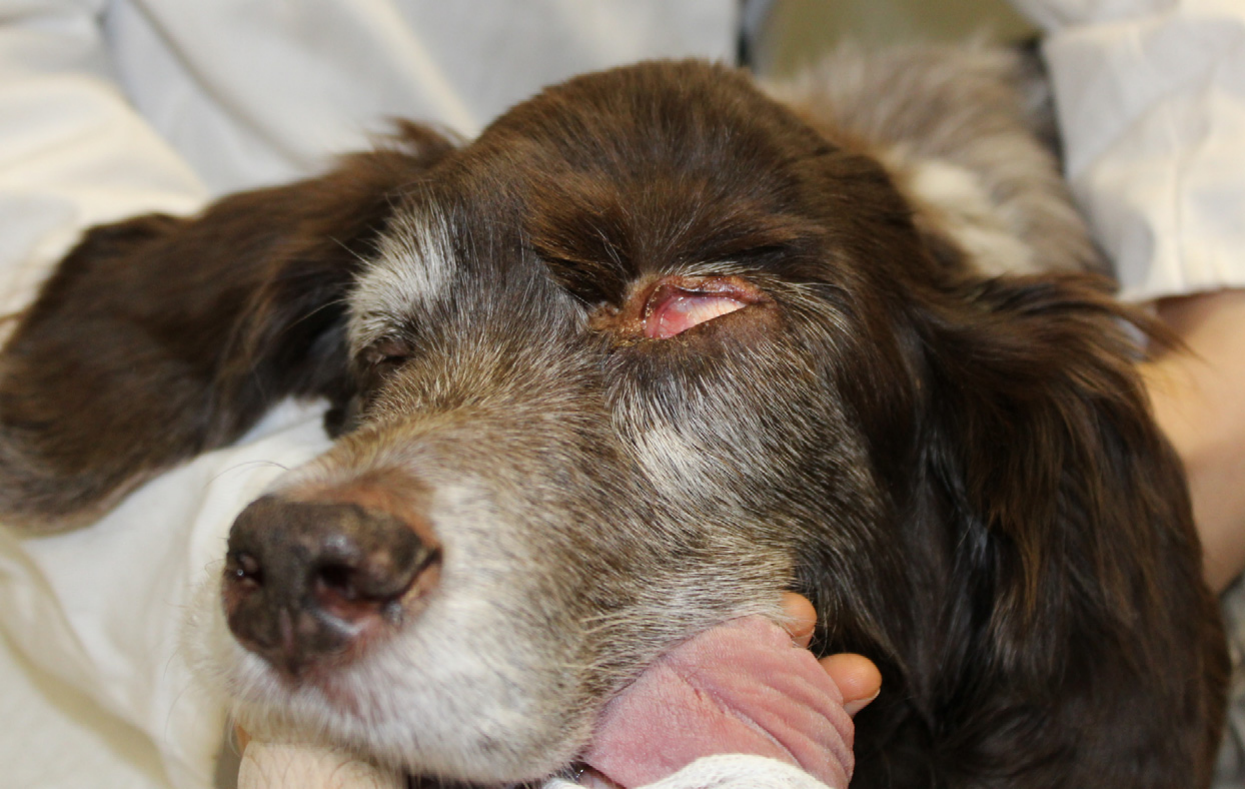
No evidence of visible tumour regrowth 17 months after surgery. Dog anaesthetized.

Only mild bloody nasal discharge was observed by the owners at this stage. No evidence of visible and palpable tumor regrowth could be found clinically (Fig 14).

Chemotherapy was stopped on the grounds of not having any therapeutic effect. Changing into any another treatment protocol (Cisplatin e.g.) was declined by the owners.

#### Outcome

The patient was discharged from the hospital due to the relative absence of symptoms. The owners were advised to euthanize the dog should clinical signs. This came to pass 21 months after orbitectomy and 5 months after finishing chemotherapy because of progressive epilepsy.

## Discussion

Human studies have shown a poor prognosis when the pFS‐SCC is advanced 1. In an old study 23 an average survival period of only 14 months was reported. The recurrence rate appears to be high. In two case reports and with different treatments, the tumors recurred between three and 14 months after surgery 1, 4. The prognosis of human patients with stage 3 and 4 pFS‐SCC is extremely poor despite the performance of radical resection followed by postoperative radiotherapy or chemotherapy 1, 4.

Recommendations for removal of pFS‐SCC in dogs do not exist. For oral SCC surgical margins of at least 2 cm are recommended 24. But in the face it is difficult to remove a tumor in the requested margins. Goodger and Carlson (1972) removed an mucinous adenoma and undifferentiated carcinoma after removal of the complete frontal bone in a dog 25.

If the bony orbit is involved, then an orbitectomy is necessary for curative resection 26.

Orbitectomy is infrequently reported in the veterinary literature and can be subdivided into total or partial orbitectomy. A partial orbitectomy includes an excision of either the superior (caudomedial) or inferior (rostrolateral) orbit. In case of total orbitectomy enucleation was always included 13.

In the dog in this case report the tumor extended into the orbit, causing mild proptosis. Thus in the presented case a superior orbitectomy was supposed to maximize the probability of removing all cancer cells. En bloc resection of the tumor for radical resection was performed, with preserving of the left globe. The histopathological examination revealed clean wound margins.

In one case report the pFS‐SCC could be removed, but the removal was incomplete 18. The long period of remission and the tumor free margins tell for complete removal of the tumor in the case of this report. Nevertheless local recurrence had to be noted twelve months after surgery. At this time chemotherapy was discussed and planned. Chemotherapy with carboplatin as part of a multiagent approach is a useful treatment option for oral nontonsillar SCC in dogs 27. In one study pFS‐SCC in three dogs were medically treated with piroxicam combined with carboplatin or toceranib 16: Dog 1 received carboplatin and achieved a complete remission, but was euthanized 344 days after start of therapy. Dog 2, still alive 3 years after start of therapy received 14 carboplatin deliveries. In dog 3, after changing the treatment protocol into piroxicam‐toceranib, a significant tumor reduction occurred, but the dog was euthanized after 195 days because of a relapse. In the present case the survival time was 620 days. Due to the results above the author of this paper decided to use Carboplatin. The chemotherapy started late, at time of relapse and whereas a clinical steady state could be detected chemotherapy did not prevent further tumor growing how could be seen in CT. It may be possible that adjuvant chemotherapy at the time of the surgery may have prohibited or delayed tumor regrowth. Nevertheless though this particular disease carries a poor prognosis and the short survival in longer living humans, our patient enjoyed a relatively normal life with a visual eye for 21 months.

## Conclusion

Eye sparing superior orbitectomy, possibly in combination with concurrent chemotherapy seems to be an appropriate treatment option for of pFS‐SCC in dogs.

## Authorship

AS: sole author

## Conflict of Interest

None declared.
